# The complete genome of *Vibrio diabolicus* isolated from coastal waters and Pacific oysters in England

**DOI:** 10.1128/mra.01318-24

**Published:** 2025-06-09

**Authors:** David Ryder, Megan Adaway, Frederico M. Batista, Sariqa Wagley, Andy Powell

**Affiliations:** 1 Centre for Environment, Fisheries and Aquaculture Science (Cefas), Weymouth, Dorset, United Kingdom; 2 Department of Bacteriology, Animal and Plant Health Agency (APHA), Weybridge, Surrey, United Kingdom; 3 Biosciences, College of life and Environmental Sciences, University of Exeter, Devon, United Kingdom; California State University San Marcos, San Marcos, California, USA

**Keywords:** *Vibrio*, *Vibrio harveyi*

## Abstract

Water and Pacific Oyster samples were collected from an estuary in Southwest England. Primary identification of bacteria suggested isolates (n = 10) were *Vibrio alginolyticus*; however, phylogenetic analysis using whole genome sequencing Illumina and Nanopore data showed they were *Vibrio diabolicus*.

## ANNOUNCEMENT

Initially isolated from deep-sea hydrothermal fields (1), *Vibrio diabolicus*, including those isolates previously classified as *V. antiquarius*, have been found in a range of different global environments (2).

To enhance the diversity of *Vibrio* sp. in mock community experiments, samples were collected from Poole Harbor in summer 2020. Surface water samples were taken in volumes of 1 L and concentrated using 0.45 µm membrane filters. Pacific Oyster *Magallana (Crassostrea) gigas* samples were processed as described by Stockley (3), but, in brief, 10 oysters were shucked and pooled, from which 25 g was stomached for 3 min and diluted in 75 mL prewarmed (37°C) alkaline salt peptone water (ASPW, CM1117B). The sample was stomached for a further 3 min and transferred to 150 mL ASPW. This was repeated, and then samples were incubated at 37°C ± 1°C and 41.5°C ± 1°C for 6 h. Following filtration of seawater samples and enrichment of bacteria from oysters, subculturing followed a single approach whereby material was transferred onto thiosulfate citrate bile sucrose agar (TCBS Oxoid CM0333B) and ChromID Vibrio (VID) (Biomerieux) and incubated at 37°C ± 1°C for 24 ± 3 h. Plates were examined, and single colonies were picked based on morphology (size, color, shape, smell). Colonies were purified on Marine Agar (MA) 37°C ± 1°C for 24 ± 3 h (BD DIFCO Marine Agar 2216) ready for DNA extraction.

Colonies were direct plated onto MBT Biotarget 96 plates in duplicate, including an *Escherichia coli* standard, and overlaid with 1 µL of HCCA Matrix (Bruker). After drying, plates were transferred to a MALDI Biotyper Sirius (Bruker Daltonik GmbH) and run in positive linear ion mode, between 1,800 and 20,200 Da and with 8,000 shots per spot. MBT Compass HT was used to compare against reference spectra. Samples were assigned to *V. alginolyticus* with log confidence scores > 2.0.

Bacterial DNA was extracted from 10 isolates using the Maxwell extraction robot (Promega) following instructions provided for the Maxwell RSC cultured cell DNA kit (Promega). The quantity of DNA was determined using Glowmax (Promega). Illumina sequencing was performed by the Earlham Institute using Nextera XT libraries run on a NovaSeq 6000 (2 × 250 bp). A nanopore sequencing library was prepared using the SQK-LSK112 kit following an online protocol (4). Sequencing was done on a MK1C running MinKNOW 21.05.25 and a FLO-MIN112 R10.4 flowcell (read N50: 3,470 bp).

Illumina reads were trimmed using BBMap v38.75 (parameters: ktrim = r k = 21 mink = 11 hdist = 2 tpe tbo) (5) and assembled using SKESA version 2.4.0 (6).

Nanopore reads were basecalled using Guppy 5.0.16 and the super-accuracy model and processed using the split_on_adapter tool in duplex-tools v0.2.7 (7). Trycycler v0.5.4 (8) was used to create 12 independently subsampled read sets (parameters: --min_read_depth 40). Read sets were assembled using three different approaches: flye 2.9.3 (9) (parameters: --read-error 0.05 --nano-hq); minimap 2.26 (10), miniasm 0.3 (11), and minipolish 0.1.2 (12); and racon 1.5.0 (13). Trycycler v0.5.4 was used to determine the consensus across each assembly, including circularization and rotation (see reference 14). Polypolish 0.6.0 (15) and bwa 0.7.18 (16) were used to polish the genome using Illumina reads (see reference 17). An additional round of short read polishing was done using pypolca 0.3.1 (18).

Assembles were annotated using Bakta v1.9.3 and v5.1 of the full database (19). Annotated references originally submitted to NCBI as *V. antiquarius* (*n* = 20) or *V. diabolicus* (*n* = 76) were downloaded, with *V. alginolyticus* ATCC 17749 (GCF_000354175.2) and *Vibrio parahaemolyticus* RIMD 2210633 used as outgroups. PIRATE 1.0.5 (20) was used to identify the core genome of *V. diabolicus*. IQ-Tree 2.3.4 was used to construct a maximum likelihood phylogenetic tree (21, 22) (parameters: -m GTR+F+R4 -alrt 1000 -B 1000).

**Fig 1:**
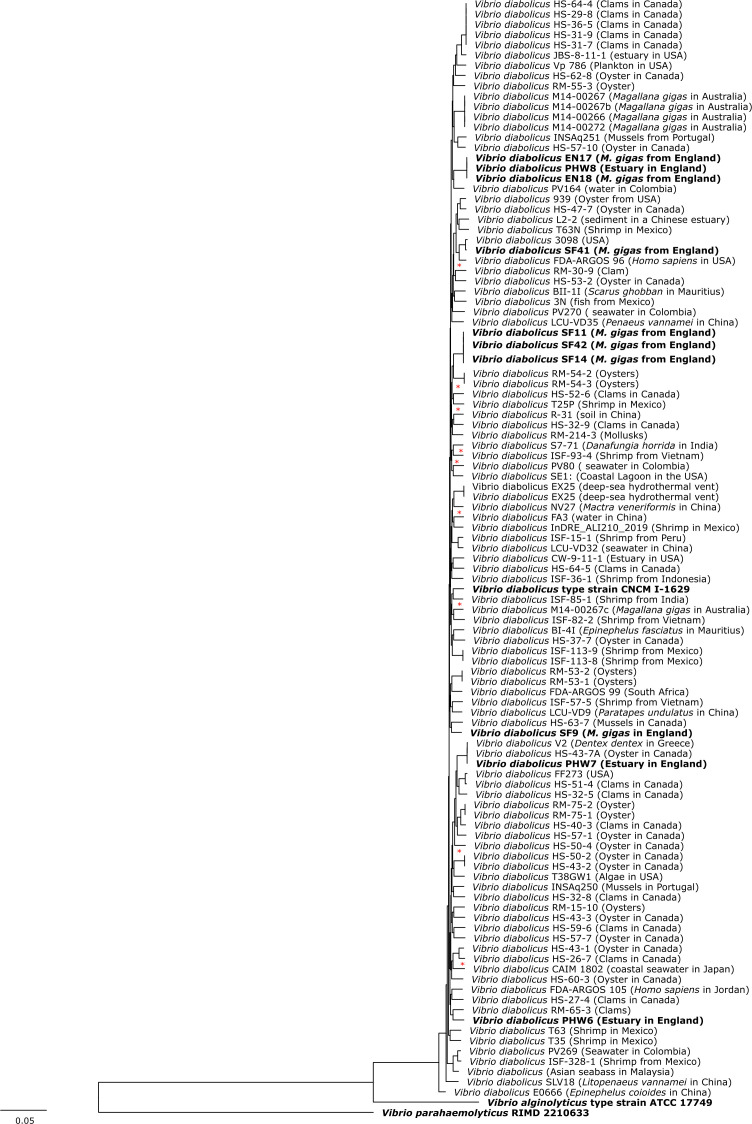
Maximum likelihood phylogenetic tree based on the *Vibrio diabolicus* core genome using a general time reversible model with four categories and FreeRate heterogeneity. Unsupported clades with less than 95% support using an ultrafast bootstrapping approach or 80% support according to the SH-aLRT test are shown with a “*”.

This announcement shows that *V. alginolyticus* isolates initially identified using primary identification techniques, such as MALDI-TOF, were in fact *V. diabolicus* based on whole genome sequencing (see Fig. 1).

**TABLE 1:** Metadata and genome assembly metrics for five *Vibrio diabolicus* isolates from water and bivalve samples collected from Poole Harbour

Isolate	Isolation source	GC (%)	Depth (x)	No. of reads	Genome size (bp)	N_50_ (kb)	No. of contigs	Coordinates	BioSample	SRA	Assembly
SF9	*Magallana gigas*	45	229	4,001,660	5,007,999	315	42	50.7°N, 2.0°W	SAMN43405760	SRR30473575	GCA_041894775.1
SF11	*M. gigas*	45	288	5,085,578	5,035,057	338	61	50.7°N, 2.0°W	SAMN43405761	SRR30473574	GCA_041894765.1
SF14	*M. gigas*	45	288	4,773,436	5,125,236	335	63	50.7°N, 2.0°W	SAMN43407143	SRR30474948	GCA_041894785.1
SF41	*M. gigas*	45	222	3,874,230	5,030,111	379	36	50.7°N, 2.0°W	SAMN43405762	SRR30473573	GCA_041894745.1
SF42 (Nanopore)	*M. gigas*	45	89	133,962	5,214,033	3,285	3	50.7°N, 2.0°W	SAMN43405763	SRR30473571	GCF_045586155.1
SF42 (Illumina)	*M. gigas*	-	197	4,195,462				50.7°N, 2.0°W		SRR30473572	
EN17	*M. gigas*	44.5	282	5,198,056	5,254,379	197	67	50.7°N, 2.0°W	SAMN43405764	SRR30473570	GCA_041894645.1
EN18	*M. gigas*	44.5	230	4,280,490	5,254,444	227	65	50.7°N, 2.0°W	SAMN43405765	SRR30473569	GCA_041894685.1
PHW6	Estuary	44.5	258	4,611,192	5,147,664	265	70	50.7°N, 2.0°W	SAMN43405766	SRR30473568	GCA_041894755.1
PHW7	Estuary	45	281	5,134,990	5,208,681	375	45	50.7°N, 2.0°W	SAMN43405767	SRR30473567	GCA_041894665.1
PHW8	Estuary	44.5	238	4,426,494	5,254,714	197	65	50.7°N, 2.0°W	SAMN43405768	SRR30473566	GCA_041894655.1

## Data Availability

Sequencing data have been deposited on NCBI under BioProject accession PRJNA1153944. Links for specific BioSample, SRA, and Assembly records for each isolate are included in Table 1. Annotations created using Bakta and used as part of the phylogenetic analysis have been uploaded to FigShare (23).
